# Network analysis of depressive symptoms in Chinese outpatients with somatic symptom disorder

**DOI:** 10.3389/fpsyt.2025.1617999

**Published:** 2025-09-03

**Authors:** Yihui Li, Junning Fang, Yunhui Zhong, Yibo Li, Yuanping Liao, Hong Tang

**Affiliations:** ^1^ Department of Psychology, Gannan Medical University, Ganzhou, Ganzhou, Jiangxi, China; ^2^ The Third People’s Hospital of Ganzhou, Ganzhou, Jiangxi, China; ^3^ Department of Psychiatry and Psychology, College of Basic Medical Sciences, Tianjin Medical University, Tianjin, China

**Keywords:** network analysis, depressive symptoms, Chinese outpatients, somatic symptom disorder, network accuracy and stability

## Abstract

**Background:**

Somatic symptom disorder and depression in clinical practice are strongly correlated. In this study, network analysis was used to assess the depressive symptoms of patients with somatic symptom disorder to identify the most core and influential symptoms. The aim of this study was to provide new perspectives for the treatment and rehabilitation of patients with somatic symptom disorder.

**Methods:**

A total of 899 individuals were enrolled from Gannan Medical University’s First Affiliated Hospital, Ganzhou People’s Hospital, and Third People’s Hospital of Ganzhou. A version of the Patient Health Questionnaire-9 was administered to assess symptoms of depression. We described the network structure of depressive symptoms, utilizing indicators of “strength,” “betweenness,” and “closeness” to identify the key symptoms within the network. A bootstrap approach with case-dropping was used to test the network’s stability.

**Results:**

Concentration (PHQ7), Motor (PHQ8), and Anhedonia (PHQ1) symptoms had the highest centrality values, the strength values are 1.67, 1.62, and 1.58 respectively. The edge connecting sad mood (PHQ2) and energy (PHQ4) were the most influential in the model, with an edge weight of 0.69, the highest among all edges.

**Conclusions:**

This network analysis study identifies distinct depressive symptomatology within the Chinese SSD patient population. Core symptoms anhedonia, cognition, and motivation primarily drive depressive symptoms, underscoring the need for clinical focus on these manifestations to prevent exacerbation. Tailored interventions targeting these core symptoms, including the integration of pleasant experiences, dopamine-based medications, attention bias modification training, and behavioral activation therapy, should be considered in treatment strategies.

## 1 Introduction

Somatic symptom disorder (SSD) or somatoform disorder (SFD) is characterized by patients exhibiting a diverse array of clinically significant symptoms that lack a clear medical explanation. These often manifest as notable physical symptoms accompanied by considerable levels of pain, discomfort, and functional impairment (1). The prevalence of medically unexplained symptoms and SSD within primary care settings varies widely, ranging from 5–35% (2). The incidence rate of somatic disorders in the general population is estimated to be 5% to 7% (3), and about 20% to 25% of patients with acute somatic symptoms will develop into chronic somatic diseases (4). Meanwhile, women are more likely than men to exhibit physical symptoms and disorders, with a male to female ratio of approximately 1:10 (1). According to epidemiological research in China, the prevalence of SSD is 0.277% in the general Chinese population (5). In another study on Chinese general hospitals, the incidence of SSD was 33.8% among patients in six outpatient departments in five cities (6).

Depressive symptoms, accompanied by inexplicable somatic complaints, significantly contribute to the burden of somatic symptom disorder (SSD) (7). Although not all patients fulfill the criteria for comorbid depression, many with SSD report core depressive symptoms such as fatigue (8), excessive guilt (9), concentration difficulties (10), and sensitivity to negative emotions, suggesting a high prevalence of depressive mood (11). This association is often enduring. Persistent pain, discomfort, and physical limitations act as direct stressors, fostering feelings of helplessness, frustration, and pessimism (12). Unexplained symptoms often lead to confusion and fear, prompting catastrophizing thoughts (13). This negative cognitive appraisal links somatic and depressive symptoms. Impaired work, social, and family roles due to somatic symptoms reduce activities and social connections, leading to a sense of loss and diminished self-worth (14), further perpetuating depressive symptoms. To prevent the worsening of these symptoms and their progression to comorbid depression, vigilance regarding depressive manifestations in SSD patients is crucial alongside basic treatment.

Evidence on the strong association between somatic symptoms and depressive symptoms is ample (15). The utilization of standardized scales to evaluate depressive symptoms is common in research. For instance, the Patient Health Questionnaire-9 (PHQ-9) (16) revealed that patients with SSD showed higher-than-average levels of depression, highlighting the importance of this approach. However, the literature suggests that distinct depressed symptoms may result in different adverse events, risk profiles, and underlying brain mechanisms (17, 18). In therapeutic settings, a single depressive symptom may be a predictor of future changes in therapy for additional symptoms. Therefore, it is important to evaluate specific depressive symptoms among patients with SSDs to provide scientific support for their treatment and rehabilitation. Network analysis (19), as an emerging statistical method, can support research on the relationships between specific symptoms.

According to a newly proposed theory of psychopathology (20) referred to as the causal system perspective of mental disorders, rather than originating from a common cause, the cluster of co-occurring symptoms of depression is thought to be secondary to direct symptom-to-symptom interactions. Different depressive symptoms interact with each other through distinct psychological and biological mechanisms, through network analysis, these symptom-to-symptom linkages can be examined. Network analysis has revealed the presence and specificity of these relationships, furthermore, core symptoms may play a major role in the onset and persistence of other symptoms. As a result, concentrating on biopsychosocial factors while addressing these core symptoms might be a better approach (21). Neighboring symptoms in network theory can activate each other, and external circumstances, such as major negative life experiences or physical problems, can also activate them (22). Consequently, the network perspective can potentially provide more clinically relevant insights into the role that early symptoms play in predicting the likelihood of future disorders. Using network analysis to reveal the association patterns between depressive symptoms in patients with SSD can help identify the most central depressive symptoms and how these symptoms interact with each other and influence SSD. In clinical practice, little attention has been paid to the depressive symptoms of patients with somatic disorders. However, due to their close relationship, the occurrence of depressive symptoms can sometimes affect the normal treatment of patients. Therefore, understanding of the depressive symptoms of patients can provide new ideas for clinical treatment.

To date, network analysis research on depressive symptoms has involved many populations, such as adults with PTSD (23), patients admitted to an interdisciplinary chronic pain (24), and clinically stable adolescents with major psychological disorders (25). Studies on Wuhan residents’ depressive symptom network during the later stages of the COVID-19 pandemic in China have been published (26). However, the results of different subjects across studies cannot be generalized. Although the correlation between somatic symptoms and depressive symptoms has been confirmed (15), network analysis research on depressive symptoms in patients with SSD is lacking. This age group experiences a high prevalence of SSD alongside substantial social and familial pressures. Although individuals over 60 also exhibit high SSD rates, their exclusion is due to potential confounding effects of age-related physiological decline on depressive symptoms. By examining depressive symptoms linked to somatic manifestations, constructing symptom networks, and identifying core symptoms, this research aims to inform rehabilitation strategies and improve psychological resilience in this key population.

## 2 Materials and methods

### 2.1 Study design and participants

Participant recruitment and data collection for this study were exclusively carried out in China to ensure operational feasibility and prompt data acquisition. A primary aim of this study is to offer empirical evidence derived from local data to clinical practitioners in China. By elucidating the online manifestations of depressive symptoms in Chinese individuals with SSD, our goal is to enhance psychological assessment, diagnostics, and treatment strategies for this population in China, offering valuable insights.

A cross-sectional survey at the First Affiliated Hospital of Gannan Medical University, the Third People’s Hospital of Ganzhou, and the Ganzhou People’s Hospital in Jiangxi Province, China, was conducted between January 2023 and April 2024. The study design was approved by the Third People’s Hospital of Ganzhou’s Institutional Review Board (IRB). Before participating in this trial, all patients provided their informed consent. The responders’ information was confidential.

A total of 1068 volunteers from Gannan Medical University’s First Affiliated Hospital, Ganzhou People’s Hospital, and Third People’s Hospital in Ganzhou participated, all selected subjects were primarily diagnosed with somatic symptoms disorder, and the occurrence of depressive symptoms before somatic symptoms was excluded in advance. For eligibility to participate, individuals needed to meet four requirements for inclusion in the study, evaluated based on the participants’ self-reporting: (1) Chinese Han nationality; (2) age range, 18–60 years; (3) SSD diagnosis according to the Diagnostic and Statistical Manual of Mental Disorders (DSM-5) criteria; (4) capacity to provide written informed consent. A supervisory senior physician with years of clinical expertise cross-checked the diagnoses before confirming them during our weekly team meeting. A total of 997 patients met the inclusion criteria, and 98 patients were excluded due to the following reasons: (1) pregnant or lactating mothers (n=23); (2) substance use disorder (n=25); (3) severe personality disorder (n= 13); (4) severe physical diseases (n=12); (5) refusal to participate in the study (n=20), and (6) due to physical reasons, the assessment was forced to be interrupted (n=5).

### 2.2 Measurements

The nine-item Chinese version of PHQ-9 (27), which evaluates cognitive, emotional, physical, and interpersonal symptoms associated with depression, was used to assess depressive symptoms. These symptoms include anhedonia, depressed mood, sleep disturbances, appetite changes, low energy, feelings of guilt, concentration difficulties, motor agitation or retardation, and suicidal thoughts within the past two weeks (28). Each item is scored from (not at all) to 3 (almost every day), with higher total scores indicating more severe depressive symptoms (mild: 5–9, moderate: 10–14, severe: ≥15). The PHQ-9’s validity has been well-established in Chinese populations (29, 30) and is confirmed for measuring mood, anxiety, personality, and psychotic disorders (31). Additionally, it demonstrates consistent measurement across different gender, racial, and educational groups (32). The Cronbach’s alpha coefficient for this study was 0.973, indicating high internal consistency among the participants.

### 2.3 Network estimation

For every PHQ-9 item, the mean, SD, skewness, and kurtosis were calculated. Network methodology characterized individual depression symptoms as “nodes,” and the relationships between these symptoms as “edges.” In the network representation, the direction of correlations is shown as the color of the edges; red and blue edges showed negative and positive correlations, respectively. The thickness of the edges represents the intensity of links between nodes (33).

Using the ‘bootnet’ package (34), which builds upon the ‘glasso’ algorithm from the ‘glasso’ package (35), we implemented the ‘EBICglasso’ method of the ‘qgraph’ package (33). Regularized partial correlation was used to assess the network architecture.

After controlling for all other variables, coefficients representing the association between two nodes ranged from -1–1. A weighted network structure was used to show these partial correlations. Each node represented a variable (such as a symptom), and each edge showed that the two variables were not independent when all other factors were considered. Their partial correlation coefficients are represented by the weights of the edges. Spearman’s correlations were used to construct a covariance matrix because the data were ordinal (36). The resulting covariance matrix was then input in the ‘EBICglasso’ algorithm, which creates sparse networks by applying the least absolute shrinkage and selection operator (LASSO) regularization (37). This approach seeks to minimize and clarify the edges in the network by using LASSO to set small correlations to zero. A substantial sample size is necessary to simplify the model, ensuring the network diagram highlights the most representative nodes and correlations, thus reducing false associations. The extended Bayesian information criterion (EBIC) is minimized by the LASSO tuning hyperparameter (λ) chosen from the ‘EBICglasso’ technique (38), where the EBIC hyperparameter (γ) was set at 0.5.

Three key centrality indices, namely betweenness, closeness, and strength, were computed to examine the most prominent symptoms in the depressive symptom network (39). Strength indicates each node’s total sum of edge weights, highlighting the significance of specific elements. Closeness is the inverse of the cumulative distance between a node and every other node in the network. Betweenness quantifies the frequency with which a node is on the shortest paths linking other nodes (40).

### 2.4 Estimation of network accuracy and stability

Three approaches were utilized to appraise the correctness and stability of the network model to determine the resilience of the system (41). Using a non-parametric bootstrapping method, we first evaluated the precision of edge weights by computing confidence intervals (CIs) (42). This required randomly resampling the observations to create new datasets from which 95% CIs were calculated. Narrower CIs suggested a more dependable network structure, while wider CIs showed lesser accuracy in edge estimations (41). Subset bootstrapping was employed to evaluate the stability of centrality indices, including betweenness, closeness, and strength, using the correlation stability coefficient (CS-C) in the second step (43). If, after removing some sample nodes, the centrality indices of those nodes showed the least possible variation, the network topology was considered stable. CS-C denoted the highest percentage of samples that could be eliminated with a minimum of 95% certainty that the correlation between the initial centrality indices would cross the 0.7 threshold (41). Ideally, CS-C values should surpass 0.5 and typically equal at least 0.25. If the 95% non-parametric CIs obtained from 2000 bootstrap iterations did not cover zero, a significant difference between the two strength indices was determined. We used bootstrapped difference tests to evaluate differences in the attributes of the network (41). To determine whether there were statistically significant differences between two edge weights or two node centrality indices, the corresponding 95% CIs were estimated. The R package “bootnet” was used for all analyses (41).

### 2.5 Association between symptom mean levels, variability, and centrality index

The associations between centrality indices and the mean scores of certain PHQ-9 items, as well as the corresponding SD values, were evaluated using Spearman’s rank-order correlation (44). Using the association between centrality indices and mean PHQ-9 item scores, the most central symptoms were compared to the most severe manifestations. The assessment of the link between centrality indices and SD was to determine if different item variability levels could be responsible for the observed centrality of symptoms (17).

## 3 Results

### 3.1 Descriptive statistics

Specifically, 320 men and 579 women, that is a total of 899 participants, fulfilled the study’s inclusion requirements. **Table 1** shows the fundamental sociodemographic details of the individuals. Among the 899 participants, 597 had an educational background below bachelor’s degree, accounting for 66.4%, while 302 had bachelor’s degree or above, accounting for 33.6%. 608 were married, accounting for 67.6%, and 291 were unmarried, accounting for 32.4%. The average age was 34.44 ± 12.37 years old. The average total score of PHQ-9 was 11.59 ± 8.28 points. As determined using the PHQ-9, **Table 2** presents the mean, SD, skewness, and kurtosis of the depressive symptoms. The average range of the 9 items is 0.92 ± 1.25-1.57 ± 0.86; the skewness range is -0.3-0.74, and the kurtosis range is -1.55- -0.43, all of which are non-normal distributions; the zero-score rates of the 9 items are 46.6%, 32.9%, 9.6%, 33.3%, 12.6%, 24.9%, 8.8%, 14.9%, and 63.1% respectively. The items with the highest mean ratings were Guilt (PHQ6) and Concentration (PHQ7), while those with the lowest mean ratings were Anhedonia (PHQ1) and Suicide (PHQ9).

**Table 1:** Socio-demographic characteristics of the study population (N = 899).

Variables	*N*	*%*
Gender		
Men	320	35.6
Women	579	64.4
Education level		
Below undergraduate^a^	597	66.4
Undergraduate or higher	302	33.6
Marital Status		
Married	608	67.6
Unmarried	291	32.4
	***Mean***	***SD***
Age (years)	34.44	12.37
PHQ-9 total score	11.59	8.28

SD, standard deviation, PHQ-9 the 9-item Patient Health Questionnaire; MEAN, average number.

a Below undergraduate = less than 12 years of education.

**Table 2:** Mean, standard deviation, minimum, maximum, skewness, and kurtosis, and frequency of depressive symptoms as measured by the PHQ-9 (N = 899).

Depressive symptoms	PHQ-9 item	M	>SD	Min	Max	Skewness	Kurtosis	% (Absence)	% (Presence)
Anhedonia	1	1.17	1.18	0	3	0.26	-1.55	46.6	53.4
Sad Mood	2	1.18	1.06	0	3	0.44	-1.04	32.9	67.1
Sleep	3	1.41	0.86	0	3	0.54	-0.46	9.6	90.4
Energy	4	1.25	1.02	0	3	0.02	-1.32	33.3	66.7
Appetite	5	1.35	0.89	0	3	0.58	-0.43	12.6	87.4
Guilt	6	1.42	1.03	0	3	-0.3	-1.17	24.9	75.1
Concentration	7	1.57	0.86	0	3	0.12	-0.70	8.8	91.2
Motor	8	1.33	0.91	0	3	0.53	-0.53	14.9	85.1
Suicide	9	0.92	1.25	0	3	0.74	-1.25	63.1	36.9

M, mean; Min, minimum; Max, maximum; PHQ-9, The Patient Health Questionnaire-9; SD, standard deviation.

### 3.2 Network structure and centrality measures analysis

**Figure 1:**
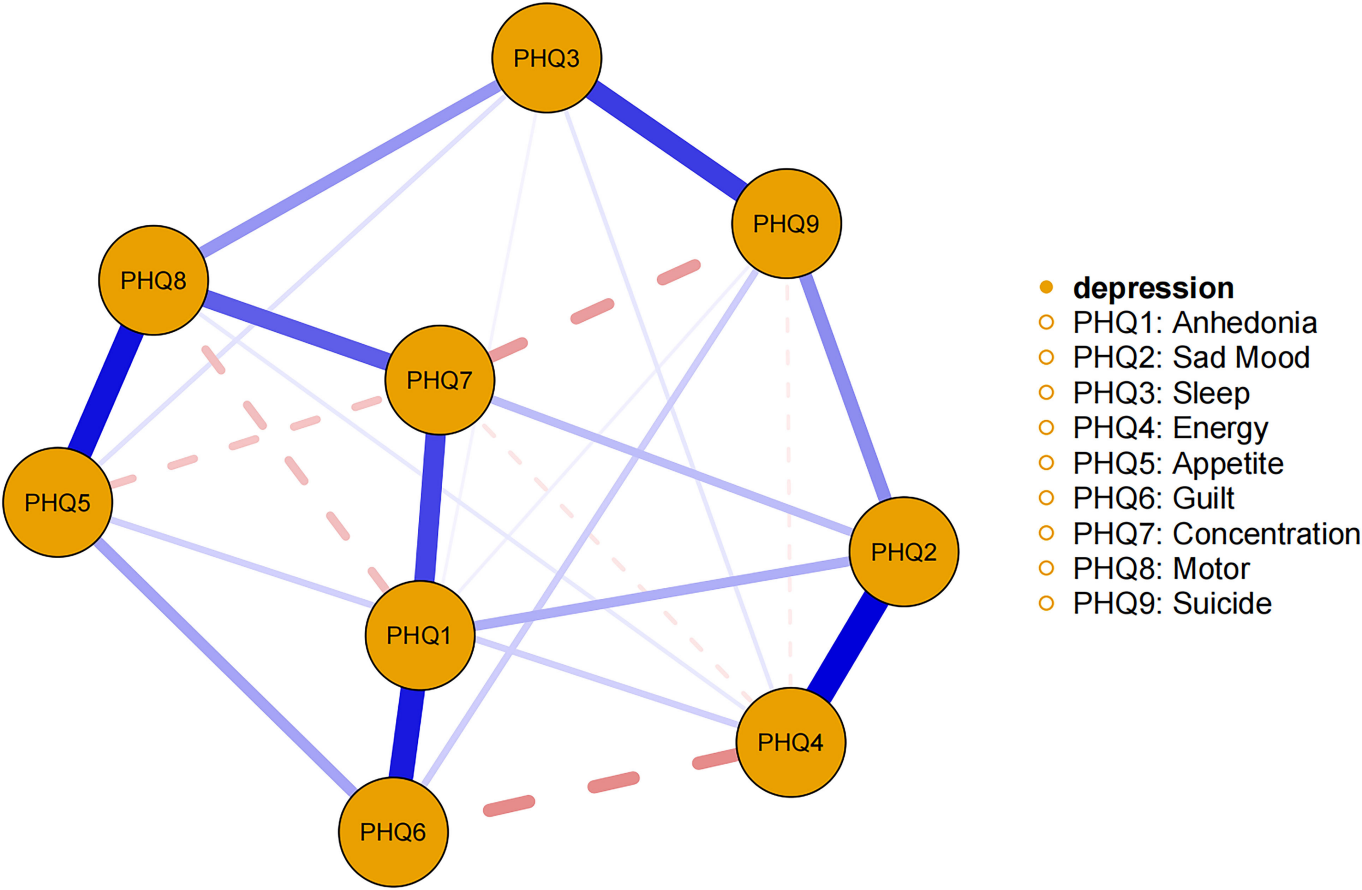
Estimated network model for dichotomized depressive symptoms in the total sample. The network model was estimated using the EBICglasso model.

**Figure 2:**
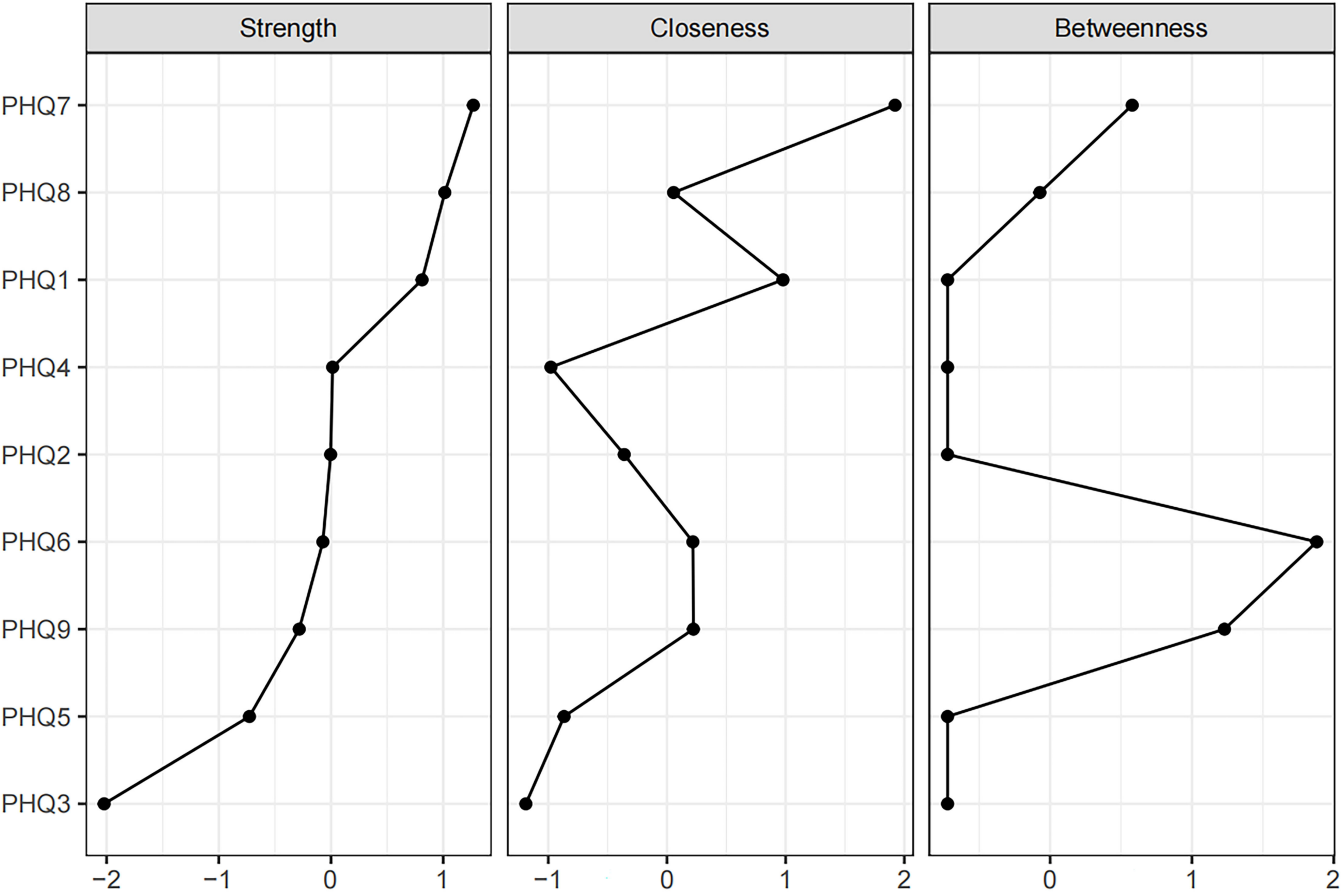
Centrality measures of all symptoms within the network. The figure shows centrality measure (i.e., strength, betweenness, and closeness) of all factors within the network (z-scores).

The network of depressive symptoms estimated using the EBICglasso algorithm, is illustrated in **Figure 1**. Several nodes exhibited strong connectivity within the network, including the strength value of 1.67 for Concentration (PHQ7), 1.62 for Motor (PHQ8), and 1.58 for Anhedonia (PHQ1). Notably, the following symptom pairs showed robust positive correlations: Appetite (PHQ5)-Motor (PHQ8), Guilt (PHQ6)-Anhedonia (PHQ1), Concentration (PHQ7)-Anhedonia (PHQ1), Sad Mood (PHQ2)-Energy (PHQ4), Concentration (PHQ7)-Motor (PHQ8), and Sleep (PHQ3)-Suicide (PHQ9). Conversely, Concentration (PHQ7)-Suicide (PHQ9), Concentration (PHQ7)-Appetite (PHQ5), Motor (PHQ8)-Anhedonia (PHQ1), and Energy (PHQ4)-Guilt (PHQ6) showed significant negative correlations. **Figure 2** presents centrality measures, strength, betweenness, and closeness, for all symptoms within the network. Concentration (PHQ7) demonstrated the highest strength, followed by Motor (PHQ8) and Anhedonia (PHQ1), while Concentration (PHQ7) and Anhedonia (PHQ1) exhibited the greatest closeness. Guilt (PHQ6) showed the highest betweenness.

### 3.3 Network accuracy and stability

**Figure 3:**
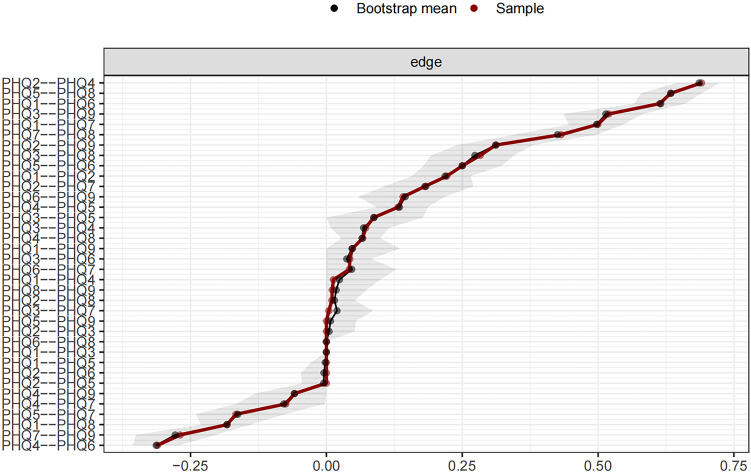
Edge accuracy plot depicting 95% confidence obtained from 2,000 bootstrap samples.

**Figure 3** shows the 95% confidence intervals (CIs) for the edge weights derived using the bootstrap approach as a gray region. Narrow CIs for these edge weights indicate a high level of accuracy in the network analysis and, overall, validate the stability of the edges measured over the network.

**Figure 4:**
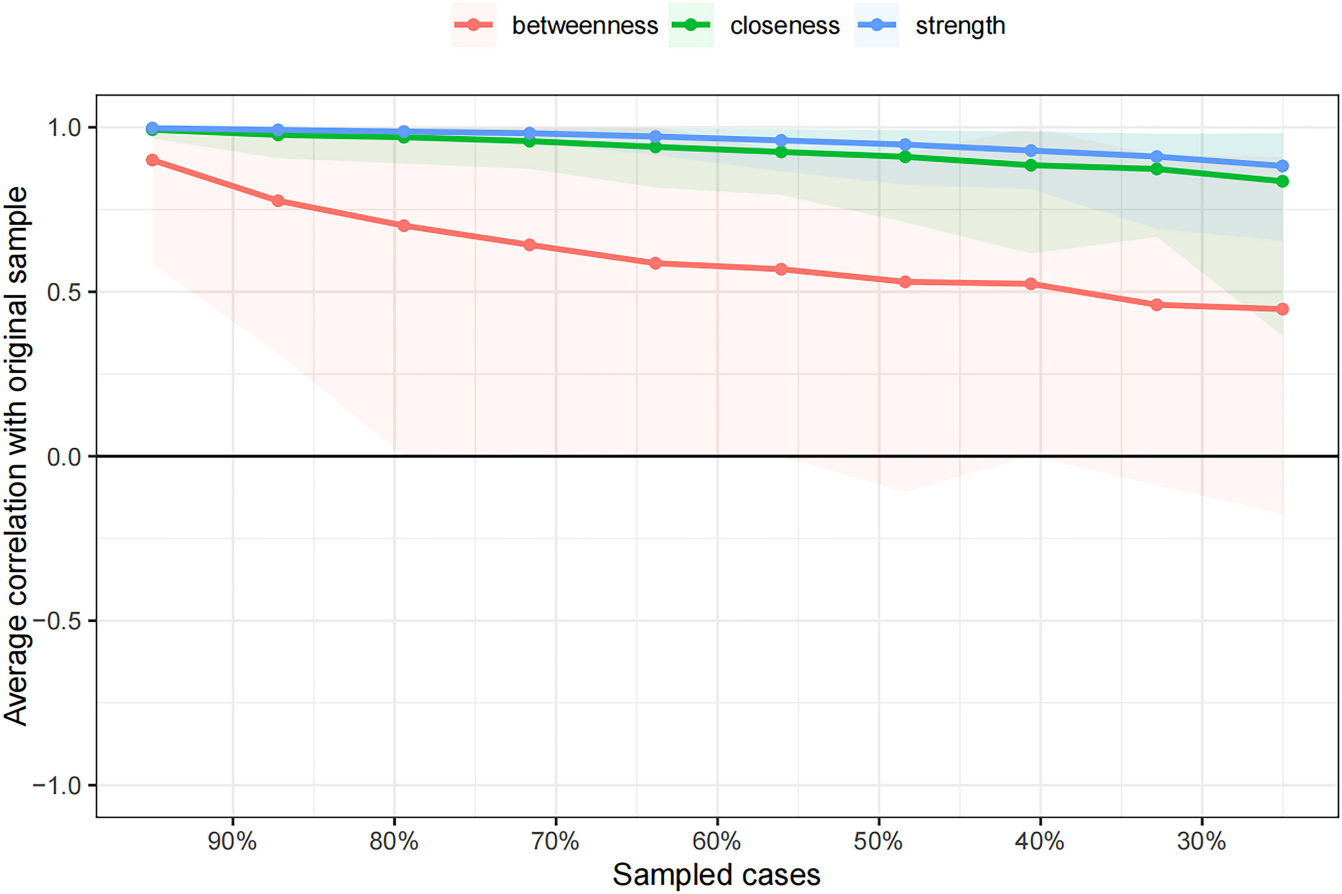
Stability of centrality indices by case dropping subset bootstrap.

The case-dropping subset bootstrap technique showed that even when significant sections of the sample were eliminated, the closeness and strength values were steady (**Figure 4**). Compared with the original study, closeness showed noticeably higher stability (CS-C = 0.672), whereas betweenness showed lesser stability (CS-C = 0.05). This sample’s strength index demonstrated robustness and reliability (CS-C = 0.75), implying that the ranking of symptoms based on strength showed a strong correlation consistent with the initial study (r = 0.7) even after eliminating as much as 75% of the sample. Thus, to describe the main symptoms in our study, we focused more on strength.

**Figure 5:**
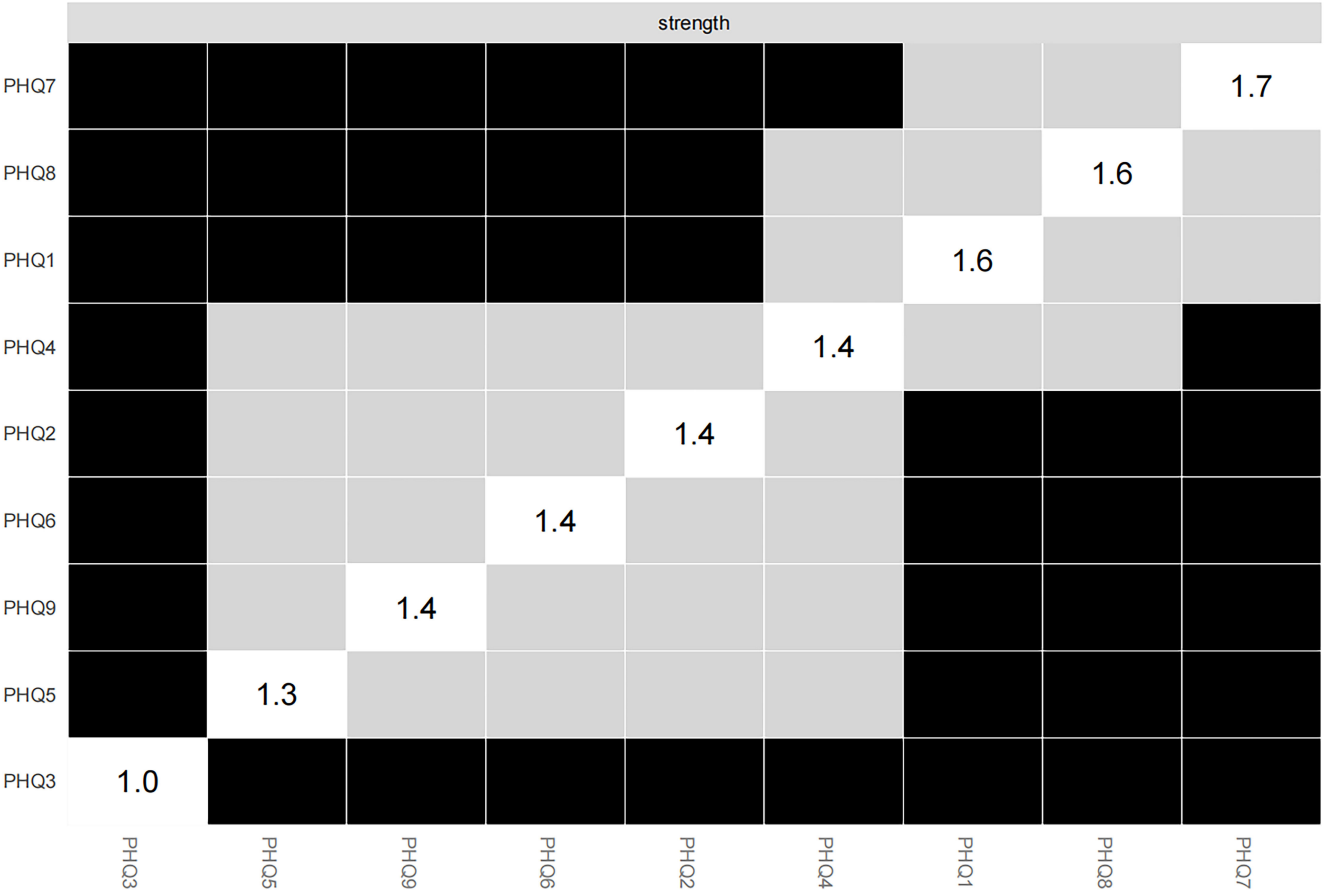
Estimation of node strength difference by bootstrapped difference test.

**Figure 6:**
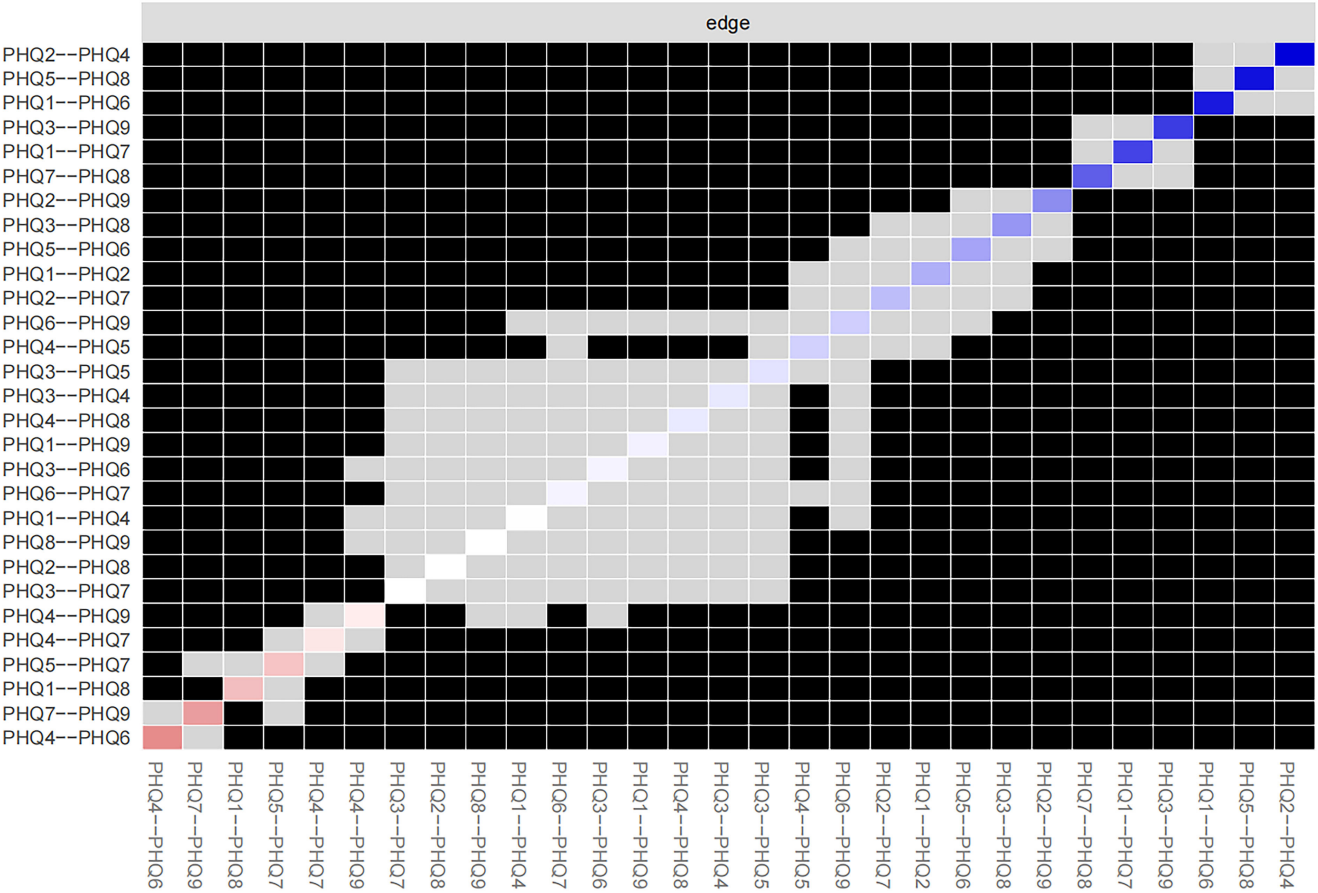
Estimation of edge weight difference by bootstrapped difference test.

Concentration (PHQ7) showed the most significant symptom strength. Additionally, both Motor (PHQ8) and Anhedonia (PHQ1) exhibited greater strength within the network (**Figure 5**). Notably, the edge linking Sad Mood (PHQ2) and Energy (PHQ4) was the most influential connection in the model (**Figure 6**), with an edge weight of 0.69, the highest among all edges.

### 3.4 Symptom mean levels, variability, and association with strength centrality index

In the overall sample, Concentration (PHQ7), Guilt (PHQ6), Sleep (PHQ3), and Appetite (PHQ5) were the depressive symptoms with the highest mean levels (**Table 2**). However, the mean PHQ-9 symptom levels exhibited no significant correlation with symptom strength (rs = 0.05). Likewise, the SD of symptoms showed no relationship with symptom strength (rs = 0.00). These findings suggest that high symptom centrality is independent of mean levels and the variability of the symptoms.

## 4 Discussion

This study investigates the independent impact of depressive symptoms in patients with SSD, rather than the link between specific physical symptoms and depressive states. It examines the interactions among depressive symptoms in Chinese patients attending SSD clinics, using network analysis models to pinpoint key symptoms with core driving effects, thus providing targets for precise psychological intervention. By focusing on the depressive symptom network, the study identifies, for the first time, the core symptoms driving depressive manifestations in Chinese outpatient SSD patients. It delineates a distinct depressive symptom network, with Concentration (PHQ7), Motor (PHQ8), and Anhedonia (PHQ1) showing the highest centrality. These symptoms serve as pivotal drivers that exacerbate or sustain other depressive manifestations. Notably, the strongest edge connectivity is observed between Sad Mood (PHQ2) and Energy (PHQ4).

Concentration (PHQ7) problem was the most central depressive symptom in Chinese outpatients with SSDs. Concentration is the ability to focus one’s attention on a particular event or activity. It is an important component of the cognitive function. Previous studies have confirmed impaired cognitive function, including concentration, caused by SSD (10). These findings are consistent with our results. Similar findings have been reported in previous studies on depressive symptom networks among adults with PTSD (23), patients admitted with interdisciplinary chronic pain (24), and those with depression and anxiety after cognitive behavioral therapy (45). However, some studies do not support problems in concentration as the core of depressive symptoms (22, 46, 47). We speculate that these differences are due to variations in the participant characteristics. Compared with the general population and student participants, patients with mental health disorders are more likely to experience concentration difficulties. Specific symptoms of patients with SSDs, such as headache, back pain, chest pain, arms/legs pain, and abdominal pain, as well as specific autonomic symptoms, such as decreased or increased appetite, may cause concentration problems in day-to-day activities, forcing these individuals to focus on their somatic symptoms, leading to repeated examinations and treatments. The persistent monitoring of somatic sensations by individuals with somatic symptom disorder (SSD) imposes a substantial cognitive burden, resulting in diminished attentional control and cognitive resource depletion. Furthermore, the rumination associated with depression (48), coupled with patients’ preoccupation with their condition, further compromises their attention to everyday tasks. The excessive focus on somatic symptoms often elicits various negative emotions, thereby exacerbating other depressive symptoms.

Motor (PHQ8) was another prominent central symptom in the network of depressive symptoms among Chinese outpatients with SSD, as indicated by its strength. Psychomotor problems manifest as psychomotor delays in depressive symptoms and refer to a significant reduction in behavioral and verbal activities. Patients with depressive symptoms usually have poor mental and motor abilities (49), and due to reduced activity, dopamine secretion decreases, leading to a more depressed mood (50). Numerous studies have supported psychomotor difficulties as among the most significant individual signs of depression (51, 52). Among them, in the Wuhan residents’ depressive symptoms network during the latter stages of the COVID-19 pandemic (26), due to the restrictions on residents’ travel and exercise caused by the epidemic, psychomotor symptoms emerged as one of the core symptoms, leading to a significant increase in their depressive mood (26). Patients with SSD often experience chronic vigilance and distress regarding their somatic symptoms, resulting in a persistent stress state. Stress activates the hypothalamic-pituitary-adrenal (HPA) axis, leading to glucocorticoid production. These glucocorticoids penetrate the brain, bind to receptors (53), and influence brain activity and behavior, thereby affecting psychomotor symptoms. Long-term high glucocorticoid levels in adulthood are linked to depression onset (53). Moreover, the sustained focus on somatic sensations depletes attentional resources, causing extreme fatigue and “lack of energy,” manifesting as psychomotor retardation. Thus, addressing psychomotor issues is crucial in clinical practice, as it may alleviate depressive and related symptoms in SSD patients. Understanding this can inform prevention and treatment strategies for comorbid depression in SSD.

In this study, Anhedonia (PHQ1) ranked third among depressive symptoms. Anhedonia is a withdrawal reaction to ongoing, unmanageable stress. It is typified by a decreased interest in or lack of pleasure from previously enjoyable activities (54). Similar findings have been reported in previous studies on depressive symptom networks among patients with prostate cancer from treatment centers in southeast Queensland (55) and clinically stable adolescents with major psychiatric disorders (25). Patients with SSD experience persistent somatic symptoms, forming a neural adaptation akin to chronic pain, which leads to reduced dopamine release (56, 57) and decreased responsiveness to pleasurable stimuli. Long term physical pain perception also leads to a decrease in social activities and entertainment measures for SSD patients, a lack of positive stimulus input, and exacerbates the lack of motivational pleasure. At the same time, excessive attention to bodily signals by patients can occupy cognitive resources, weaken the allocation of attention to pleasurable events, and may form a negative cycle of “pain- anhedonia”. According to the literature on comorbid mental illnesses (58, 59), anhedonia is a frequent and crucial link between various symptom groups. It is both a defining vulnerability of depression and a sign of depression. Focusing on the symptoms of anhedonia enables the prediction of levels of depression and somatic symptoms in patients with SSD and devising appropriate preventive measures for comorbid depression in SSD to prevent the worsening of depressive symptoms and somatic symptoms.

Viewed through a causal system perspective (20), the prominence of Concentration (PHQ7), Motor (PHQ8), and Anhedonia (PHQ1) underscores a self-perpetuating symptom network specific to individuals with somatic symptom disorder (SSD). The depletion of cognitive resources (PHQ7) directly contributes to psychomotor retardation (PHQ8) by diverting attention to bodily cues, while reduced reward processing (PHQ1) further diminishes participation in rewarding activities. This establishes a reciprocal causal relationship: motor inertia exacerbates cognitive exhaustion, which in turn intensifies anhedonia. This sequential transmission of symptoms supports the fundamental principle of the model that central elements actively trigger peripheral symptoms to sustain the persistence of the disorder.

Within the network model, the correlation between Sad Mood (PHQ2) and Energy (PHQ4) was the strongest, indicating that in this sample of patients, sad mood often corresponded to lower energy levels. Fatigue is a significant symptom of depression, characterized by reduced energy levels that contribute to increased fatigability and diminished activity (60). Somatic symptom disorder (SSD) is characterized by altered perception of bodily signals (61). Research indicates that dysregulation of the serotonin transmission system plays a critical role in SSD (62). Serotonin is pivotal in emotional regulation, and its depletion can lead to attentional biases toward negative stimuli (63), increasing negative emotions and reducing energy. This indicates a physiological connection between serotonin levels and SSD. Patients with SSD endure emotional distress from physical discomfort, which lowers their pain threshold and increases pain sensitivity (64, 65). This prolonged sensitivity intensifies fatigue, creating a “sad mood - low energy” cycle. Emotional depression further reduces interest and motivation, leading to decreased activity, as persistent negative thoughts drain psychological energy. The strong correlation between Sad Mood (PHQ2) and Energy (PHQ4) reflects the “classical depressive pattern” (66) cautioning clinicians against misinterpretation. Although these are typical depressive symptoms, patients primarily experience somatic symptoms, urging clinicians to adopt a more careful approach in diagnosis.

The Granular Interaction Thinking Theory (GITT) (67) proposes a mechanistic link between unexplained somatic symptoms and depressive networks in somatic symptom disorder (SSD) (67). According to GITT, somatic discomforts in SSD function as “physical information granules” that continuously interact with pre-existing illness schemas dominated by catastrophic interpretations. This granular interaction depletes cognitive resources through two processes: 1) somatic granules bind attentional resources, manifested as central symptoms on the PHQ-7, reducing capacity for rewarding experiences, similar to the resource depletion caused by cognitive impairment in methamphetamine addicts, and exacerbates the entropy increase of negative information processing (68); 2) repeated granule-schema interactions strengthen negative self-perceptions, leading to difficulties in information reorganization, further weakening cognitive resources and reinforcing negative loops (69), directly generating anhedonia (PHQ-1) and motor inhibition (PHQ-8). Consequently, GITT explains how SSD uniquely generates depression through cognitive-motivational pathways, in contrast to the classical affective routes. This theory elucidates the transformation of the typical affective-energy comodulation (PHQ2-PHQ4) into a pathway for cognitive-motivational symptom dominance in SSD.

This study diverges from traditional comorbidity research on somatoform disorder and depression by employing network analysis to reveal an independent network of depressive symptoms within the SSD population. Depressive symptoms are not merely adjuncts to SSD; rather, they form a distinct network centered on specific symptom nodes, influencing the emergence and progression of depressive symptoms. This network highlights characteristics unique to this sample, with depressive symptoms in the SSD population predominantly manifesting as anhedonia-cognitive-motivational symptoms, rather than typical affective symptoms like sad mood or suicidal ideation. Clinicians should note: (1) Traditional depression scale scores may miss the nuances of patients’ depressive symptoms. By closely examining patients’ responses, clinicians can better identify specific depressive characteristics. For patients with SSD, focus should be on their hedonic experience, cognitive resources, and psychomotor symptoms. (2) These findings advocate for refining treatment strategies for SSD by targeting core symptoms, building on basic pharmacological psychotherapy. For Anhedonia (PHQ1), combining dopaminergic drugs with activities that rekindle life’s pleasures (70) may reactivate the reward system. For Concentration (PHQ7), attentional bias modification training (71) can redirect focus from somatic symptoms, breaking the cycle of cognitive resource depletion. For Motor (PHQ8), behavioral activation therapy (72) can enhance motor energy and encourage movement. Given the pivotal role of core symptoms in the manifestation of depressive disorders, clinicians should heed warnings when SSD patients express feelings of “boredom,” “memory difficulties,” or “physical heaviness.” These indicators warrant careful assessment for depression risk and prompt psychological intervention to mitigate the progression and worsening of depressive symptoms.

### 4.1 Limitations

This study’s strengths include its concentrated sample specialty and substantial sample size, which are uncommon in research on patients with somatic symptom disorder (SSD). However, several limitations must be acknowledged. First, the cross-sectional design precludes establishing causation or exploring dynamic interactions between variables. Second, findings cannot be generalized to other mental health conditions, such as major depression, bipolar disorder, and personality disorders, as the study focuses on somatic symptom-related issues. Third, the use of the PHQ-9 for assessing depressive symptoms, instead of a clinical interview, may introduce bias, complicating the identification of abnormal features. This approach raises the possibility of co-occurring depression among participants, potentially increasing bias. Additionally, the influence of Berkson’s bias (73) cannot be excluded. Furthermore, detailed information on the specific manifestations and comorbidities of SSD patients was not provided. Lastly, to minimize the response burden on unpaid research volunteers, potential confounding factors like medication use and chronic conditions were not evaluated. These limitations highlight the need for further investigation in future research.

## 5 Conclusions

In conclusion, from the network analysis, the three main symptoms of depression identified in this study were Concentration (PHQ7), Motor (PHQ8) and Anhedonia (PHQ1). The “backbone” supporting the depressive symptom structure in patients with SSD includes these three primary symptoms, which provide a new perspective for clinical treatment, prompting clinical workers to pay more attention to patients’ depressive symptoms, especially core symptoms while administering routine treatment. Timely psychological health treatment and humanistic care should be provided to prevent the occurrence of comorbid depression and bring about positive effects of the treatment of SSD.

## Data Availability

The raw data supporting the conclusions of this article will be made available by the authors, without undue reservation.
